# The Phosphohistidine Phosphatase SixA Targets a Phosphotransferase System

**DOI:** 10.1128/mBio.01666-18

**Published:** 2018-11-27

**Authors:** Jane E. Schulte, Mark Goulian

**Affiliations:** a Graduate Group in Biochemistry and Molecular Biophysics, Perelman School of Medicine, University of Pennsylvania, Philadelphia, Pennsylvania, USA; b Department of Biology, University of Pennsylvania, Philadelphia, Pennsylvania, USA; c Department of Physics & Astronomy, University of Pennsylvania, Philadelphia, Pennsylvania, USA; University of Chicago

**Keywords:** CvrA, histidine phosphatase, histidine phosphorylation, PtsN, YcgO

## Abstract

SixA, a well-conserved protein found in proteobacteria, actinobacteria, and cyanobacteria, is the only reported example of a bacterial phosphohistidine phosphatase. A single protein target of SixA has been reported to date: the Escherichia coli histidine kinase ArcB. The present work analyzes an ArcB-independent growth defect of a *sixA* deletion in E. coli. A screen for suppressors, analysis of various mutants, and phosphorylation assays indicate that SixA modulates phosphorylation of the nitrogen-related phosphotransferase system (PTS^Ntr^). The PTS^Ntr^ is a widely conserved bacterial pathway that regulates diverse metabolic processes through the phosphorylation states of its protein components, EI^Ntr^, NPr, and EIIA^Ntr^, which receive phosphoryl groups on histidine residues. However, a mechanism for dephosphorylating this system has not been reported. The results presented here suggest a model in which SixA removes phosphoryl groups from the PTS^Ntr^ by acting on NPr. This work uncovers a new role for the phosphohistidine phosphatase SixA and, through factors that affect SixA expression or activity, may point to additional inputs that regulate the PTS^Ntr^.

## Abstract (precis)

One common means to regulate protein activity is through phosphorylation. Protein phosphatases exist to reverse this process, returning the protein to the unphosphorylated form. The vast majority of protein phosphatases that have been identified target phosphoserine, phosphotheronine, and phosphotyrosine. A widely conserved phosphohistidine phosphatase was identified in Escherichia coli 20 years ago but remains relatively understudied. The present work shows that this phosphatase modulates the nitrogen-related phosphotransferase system, a pathway that is regulated by nitrogen and carbon metabolism and affects diverse aspects of bacterial physiology. Until now, there was no known mechanism for removing phosphoryl groups from this pathway.

## INTRODUCTION

Phosphorylation is a common mechanism for modulating protein function. Much of its utility depends on the reversibility of the process, which is often mediated by protein phosphatases. Phosphorylation of histidine and aspartate residues is frequently encountered in bacterial regulatory systems, as well as in plants and some unicellular eukaryotes, and there is a growing appreciation for the importance of histidine phosphorylation in animal cells. However, the vast majority of phosphatases reported to date dephosphorylate serine, threonine, or tyrosine.

Escherichia coli SixA (**s**ignal **i**nhibitory factor **X**) was one of the first phosphohistidine phosphatases to be discovered and, together with the eukaryotic PGAM5 and PHPT1 (1, 2), remains one of the best characterized. Amino acid sequence features and the crystal structure of SixA show that it is a member of the histidine phosphatase superfamily (3). The name for this superfamily is derived from the conserved histidine residue that is essential for catalysis and does not reflect the substrate specificity of the superfamily’s members (4). In fact, the substrate preferences for this family of phosphatases are diverse, ranging from small phosphorylated metabolites to large phosphoproteins containing phosphorylated tyrosine, serine, threonine, or histidine residues. As the smallest member to have been crystalized, SixA’s structure is representative of the minimal core fold of the superfamily’s catalytic domain.

As of this writing, all E. coli isolates with fully sequenced genomes contain *sixA*, making it a member of the E. coli core genome. In addition, homologs of *sixA* are widespread among proteobacteria, actinobacteria, and cyanobacteria (5). Thus, SixA likely plays an important role in the physiology of these organisms. Previous work suggested that E. coli SixA dephosphorylates the histidine-containing phosphotransfer (HPt) domain of ArcB (6, 7), a histidine kinase that, together with its partner response regulator ArcA, regulates the transition to anaerobiosis. Since those initial studies, however, no additional research has been published exploring the function of SixA.

Here, we reexamine the role of SixA in E. coli. We do not observe an effect of SixA on ArcB/ArcA-regulated transcription, and we find that SixA-null strains have a growth defect that is independent of ArcB. Suppressor screens and analysis of various mutants suggest that the growth defect is due to misregulation of the nitrogen-related phosphotransferase system (PTS^Ntr^). We further show that the phosphorylation state of EIIA^Ntr^, one of the protein components of the PTS^Ntr^, is affected by SixA, and we propose a model in which SixA dephosphorylates the PTS^Ntr^ protein NPr.

## RESULTS

### The absence of SixA causes a growth defect in minimal medium.

**FIG 1:**
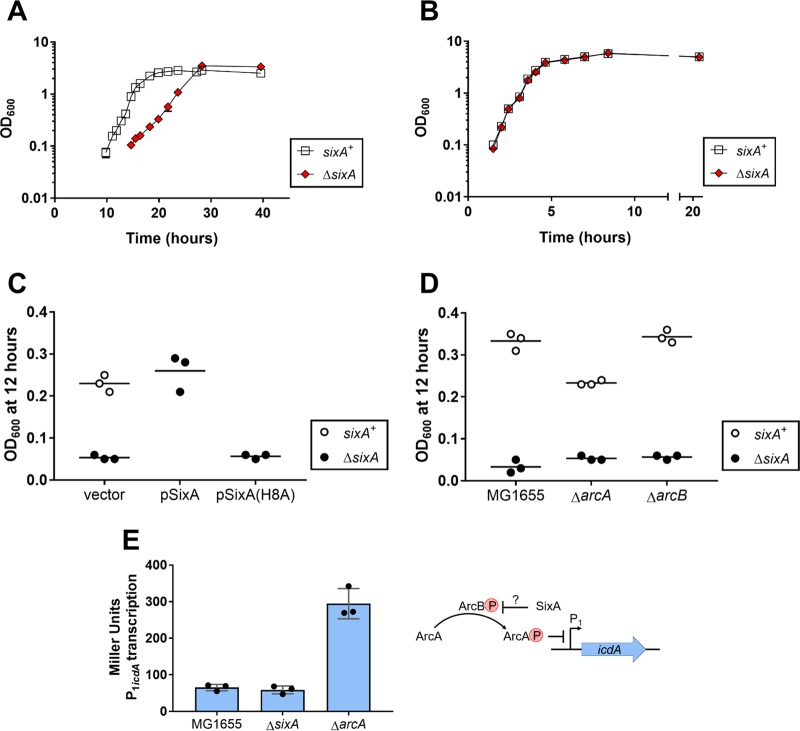
Cells lacking SixA have a growth defect that is independent of ArcB/ArcA. (A and B) Growth curves were measured for the wild-type (MG1655) and Δ*sixA* (JES13) strains in (A) glycerol minimal medium and (B) LB Miller medium. Optical density at 600 nM (OD_600_) was monitored for two independent cultures of each strain. Symbols represent the average OD_600_; bars indicate ranges and are not visible where smaller than the symbol. (C) Cultures of the wild-type (MG1655) and Δ*sixA* (JES13) strains, transformed with empty vector pTrc99a, SixA-expressing plasmid pSixA, or SixA(H8A)-expressing plasmid pSixA(H8A) were grown in glycerol minimal medium with ampicillin for 12 h. Symbols represent the OD_600_ for individual cultures, and the horizontal black lines indicate the average OD_600_ from three biological replicates. (D) Strains were grown in glycerol minimal medium for 12 h. Symbols are as described for panel C. Strains are MG1655, JES13, JES47, JES48, JES49, and JES50. (E) β-Galactosidase activity was measured for strains containing a P_1_*_icdA_*-*lacZ* reporter following anaerobic growth in minimal medium with 40 mM KNO_3_. Symbols represent the activity (Miller units) measured for individual cultures, error bars report standard deviations, and the blue bars indicate the average levels of activity from the three biological replicates. Strains are JES252, JES253, and JES254. As shown in the illustration to the right of the graph, SixA was previously proposed to dephosphorylate ArcB.

We noticed that a *sixA* deletion in Escherichia coli MG1655 grows poorly in glycerol minimal medium. In aerobic cultures at 37°C, the wild-type and Δ*sixA* strains reached similar optical densities (OD) at stationary phase, but the Δ*sixA* strain grew more slowly, with an exponential-phase-doubling time twice that of the wild-type strain (Fig. 1A). In contrast, when grown in LB Miller medium, the two strains showed no difference in growth (Fig. 1B).

To facilitate growth comparisons between strains, we used an endpoint assay in which OD was measured after 12 h, a time point that gave a large difference in OD between the wild-type and Δ*sixA* strains (Fig. 1A). With this assay, we verified that reintroducing the *sixA* gene on a plasmid restores wild-type growth (Fig. 1C). Moreover, a plasmid expressing a catalytically inactive SixA mutant, SixA(H8A) (4, 6), failed to complement the *sixA* deletion (Fig. 1C). Taken together, these results demonstrate that the growth defect is due to the absence of SixA and is likely due to the absence of SixA phosphatase activity.

We also found that the Δ*sixA* strain grows more slowly than the wild-type strain in minimal medium with acetate, maltose, or glucose as a carbon source. These observations for the Δ*sixA* mutant are consistent with the results of a chemical genomics screen that followed growth of E. coli mutants under various conditions (8). Data from that study also indicate that procaine has a more pronounced inhibitory effect on the Δ*sixA* mutant than on the wild-type strain, a result that we confirmed and also took advantage of for some genetic screens described below (see Materials and Methods).

### The Δ*sixA* mutant growth defect is independent of ArcB and ArcA.

Since SixA was previously reported to be a phosphohistidine phosphatase for the histidine kinase ArcB (6, 7), we tested whether the growth defect of a Δ*sixA* strain depends on the ArcB/ArcA two-component system. To our surprise, deletion of *sixA* produced a growth defect in strains lacking either *arcA* or *arcB* (Fig. 1D), indicating that the slow growth does not depend on ArcB/ArcA activity.

To our knowledge, there is only one published report supporting a role for SixA as an ArcB phosphatase *in vivo* (7). We therefore wanted to verify that the absence of SixA affects ArcA-dependent expression under anaerobic respiratory conditions, as previously reported (7). We tested the effect of a *sixA* deletion on transcription from the *icdA* P_1_ promoter, which is repressed by phosphorylated ArcA and is not known to be regulated by other transcription factors (9) (Fig. 1E). For anaerobic growth with nitrate as an electron acceptor, deletion of *sixA* had no effect on transcription of a P_1_*_icdA_*-*lacZ* reporter (Fig. 1E). Importantly, β-galactosidase levels for anaerobic growth in the absence of nitrate were 2-fold lower than the corresponding levels with nitrate present (30 ± 5 Miller units and 60 ± 7 Miller units, respectively), indicating that the reporter is not fully repressed and is sensitive to changes in ArcA phosphorylation under conditions of nitrate respiration. Thus, for at least our strains and assay conditions, we have no evidence that SixA affects ArcB/ArcA signaling *in vivo*.

### Suppressors of the Δ*sixA* growth defect indicate a connection between SixA and the PTS^Ntr^.

**FIG 2:**
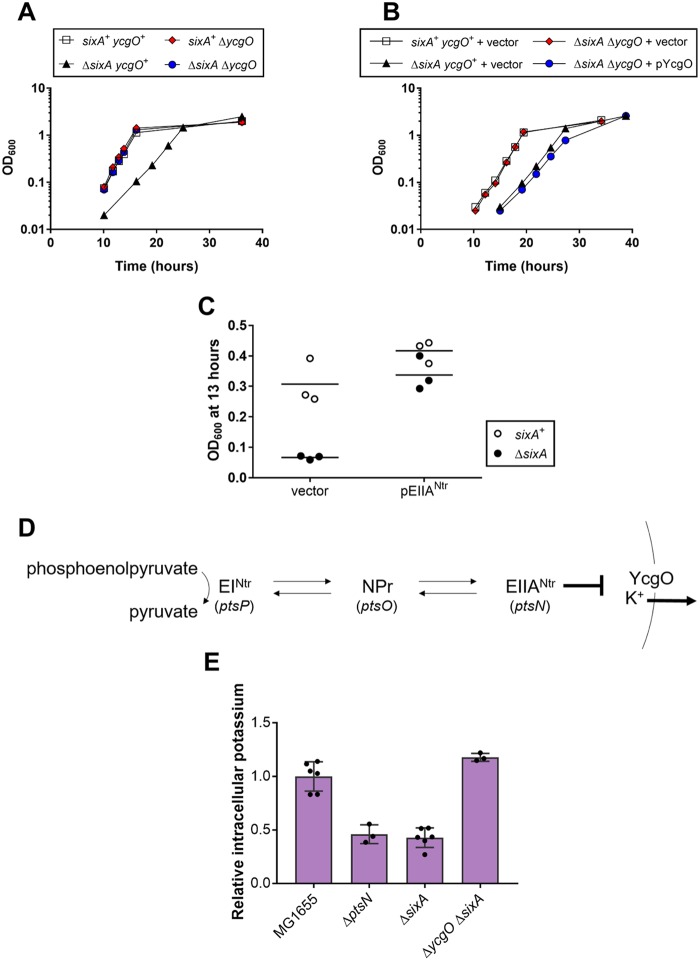
Suppressors of the Δ*sixA* growth defect. (A) Growth curves were measured for strains in glycerol minimal medium. Optical density at 600 nM (OD_600_) was measured for two independent cultures of each strain. Symbols represent the average OD_600_; bars indicate ranges and are not visible where smaller than the symbol. Samples for this growth curve were collected at the same time as the samples for the growth curve in Fig. 3B, so the data for the wild-type and Δ*sixA* strains are identical between figures. Strains are MG1655, JES13, JES30, and JES31. (B) Growth curves were measured for strains transformed with empty vector pMG91 or YcgO-expressing plasmid pYcgO in glycerol minimal medium with chloramphenicol. Symbols are as described for panel A. Strains are MG1655, JES13, and JES31. (C) Cultures of the wild-type (MG1655) and Δ*sixA* (JES13) strains, transformed with empty vector pTrc99a or *ptsN*-3×FLAG-expressing plasmid pEIIA^Ntr^, were grown in glycerol minimal medium with ampicillin for 13 h. Symbols represent the OD_600_ for individual cultures, and the horizontal black lines indicate the average OD_600_ from three biological replicates. (D) EIIA^Ntr^ is part of the nitrogen-related phosphotransferase system (PTS^Ntr^). Phosphoryl groups entering the system originate from phosphoenolpyruvate and are passed by successive phosphotransfers between the three PTS^Ntr^ proteins. According to a previously proposed model (13), unphosphorylated EIIA^Ntr^ inhibits YcgO, decreasing potassium efflux. (E) Exponential-phase cultures, grown in glycerol minimal medium, were assayed for potassium by inductively coupled plasma mass spectrometry (ICP-MS). Symbols represent the cellular potassium content for individual cultures, error bars report standard deviations, and the purple bars indicate the average cellular potassium content from all biological replicates. Strains are MG1655, JES208, JES13, and JES31. The average cellular potassium content for the wild-type strain is 243 nmol K^+^/OD_600_.

To identify genes that interact with *sixA*, we looked for mutants that suppress the slow-growth phenotype of a Δ*sixA* strain. From a pool of random transposon mutants, we identified insertions in *ycgO*, which encodes a protein sharing homology with cation/proton antiporters. We verified that a clean deletion of *ycgO* in the Δ*sixA* strain suppresses the slow-growth phenotype (Fig. 2A) and also that reintroduction of *ycgO* on a plasmid in the Δ*ycgO* Δ*sixA* strain restores a growth defect (Fig. 2B).

We also screened a plasmid library of E. coli genomic DNA to search for multicopy suppressors. As expected, the screen yielded plasmids containing *sixA*. In addition, we isolated three distinct plasmids that had only one intact gene in common: *ptsN*. Using a plasmid containing only the *ptsN* gene (modified to encode a 3×FLAG-tagged protein), we confirmed that *ptsN* overexpression suppresses the Δ*sixA* growth defect (Fig. 2C). The gene *ptsN* encodes EIIA^Ntr^, a protein of the nitrogen-related phosphotransferase system (PTS^Ntr^) (reviewed in reference 10). The PTS^Ntr^ is composed of three proteins, EI^Ntr^, NPr, and EIIA^Ntr^, and it is regulated by a series of successive phosphoryl group transfers onto histidine residues: phosphoenolpyruvate to EI^Ntr^, EI^Ntr^-P to NPr, and NPr-P to EIIA^Ntr^ (Fig. 2D). Several studies have found that inactivation of *ptsN* slows growth in minimal glucose medium (11, 12), and it was reported that deletion of *ycgO* suppresses this growth defect (13). Taken together, these observations suggest that the Δ*ptsN* and Δ*sixA* growth defects may be due to the same mechanism.

### Δ*sixA* cells have lower intracellular potassium levels than wild-type cells.

It was previously observed that cellular potassium content is lower in a Δ*ptsN* strain and is restored by deleting *ycgO* (13) (Fig. 2D). We therefore reasoned that the Δ*sixA* mutation might have a similar effect on the cell. We measured cellular potassium levels of wild-type and mutant strains by ICP-MS (13). The potassium levels of Δ*sixA* and Δ*ptsN* strains were 2-fold lower than the levels of the wild-type strain (Fig. 2E), and deletion of *ycgO* restored the potassium content of the Δ*sixA* strain to a level comparable to that of the wild-type strain (Fig. 2E). These observations suggest that the absence of SixA, similarly to the absence of EIIA^Ntr^, may cause aberrant YcgO activity and misregulation of cellular potassium content.

### Changes in SixA phosphatase activity alter EIIA^Ntr^ phosphorylation levels.

The results described above suggest a model in which the SixA phosphatase modulates phosphorylation of the PTS^Ntr^. To test this, we assayed the relative amounts of EIIA^Ntr^ phosphorylation with native gel electrophoresis, which has previously been shown to resolve the phosphorylated and unphosphorylated forms of the protein (11, 14). To detect EIIA^Ntr^ and EIIA^Ntr^-P by Western blotting, we used strains that produce 3×FLAG-tagged EIIA^Ntr^ from the native *ptsN* locus (15).

**FIG 3:**
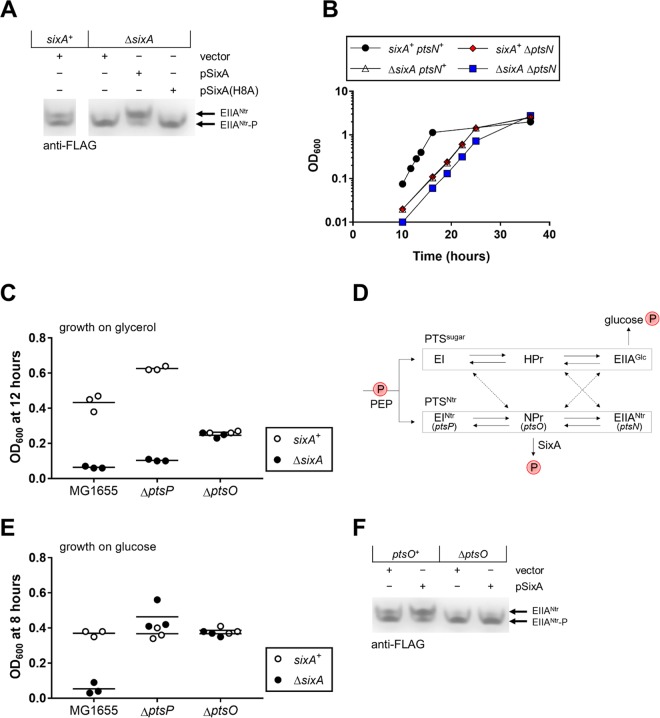
Modulation of the PTS^Ntr^ by SixA. (A) Western blot of strains expressing a 3×FLAG-tagged EIIA^Ntr^ from the native *ptsN* locus and containing empty vector pTrc99a, SixA-expressing plasmid pSixA, or SixA(H8A)-expressing plasmid pSixA(H8A). Cells were grown in LB Miller medium with ampicillin to stationary phase. Extraneous lanes from the blot are not shown. Strains are JES211 and JES212. The Western blot shown is representative of blots from three independent experiments. (B) Growth curves were measured for strains in glycerol minimal medium. Optical density at 600 nM (OD_600_) was monitored for two independent cultures of each strain. Symbols represent the average OD_600_; bars indicate ranges and are not visible where smaller than the symbol. Samples for this growth curve were collected at the same time as samples for the growth curve in Fig. 2A, so the data for the wild-type and Δ*sixA* strains are identical between figures. Strains are MG1655, JES13, JES208, and JES264. (C) Strains were grown in glycerol minimal medium for 12 h. Symbols represent the OD_600_ for individual cultures, and the horizontal black lines indicate the average OD_600_ from three biological replicates. Strains are MG1655, JES13, JES189, JES190, JES185, and JES186. (D) Schematic showing the phosphoryl group transfers within the PTS^Ntr^, potential cross-phosphorylation from the PTS^sugar^, and proposed dephosphorylation by SixA. Phosphoryl groups from phosphoenolpyruvate (PEP) are passed by successive phosphotransfers between proteins in the PTS^sugar^ and the PTS^Ntr^. Dotted arrows show cross talk between the PTS^sugar^ and the PTS^Ntr^ that has been reported to occur either *in vitro* or *in vivo* in some mutant backgrounds. Whereas the PTS^sugar^ ultimately uses phosphoryl group transfer to facilitate uptake of carbohydrates such as glucose, there are no known phosphoryl group acceptors for the PTS^Ntr^. We propose that SixA dephosphorylates NPr, providing a phosphoryl group sink for the PTS^Ntr^. (E) Strains were grown in glucose minimal medium for 8 h. Symbols are as described for panel C. Strains are MG1655, JES13, JES189, JES190, JES185, and JES186. (F) Western blot of strains expressing a 3×FLAG-tagged EIIA^Ntr^ from the native *ptsN* locus and containing empty vector pTrc99a or SixA-expressing plasmid pSixA. Cultures were grown in LB Miller medium with ampicillin to stationary phase. Strains are JES211 and JES271. The Western blot shown is representative of blots from three independent experiments.

Comparing *sixA*^+^ and Δ*sixA* strains transformed with an empty vector and grown to stationary phase in LB medium, we found that the levels of phosphorylated and unphosphorylated EIIA^Ntr^ were similar in the *sixA*^+^ strain whereas EIIA^Ntr^ was predominantly phosphorylated in the Δ*sixA* strain (Fig. 3A, lanes 1 and 2). In addition, expression of wild-type SixA from a plasmid decreased EIIA^Ntr^-P levels and increased EIIA^Ntr^ levels relative to the empty vector control (Fig. 3A, lanes 2 and 3). Finally, expression of the catalytic-site mutant SixA(H8A) did not change the relative abundances of EIIA^Ntr^ and EIIA^Ntr^-P (Fig. 3A, lanes 2 and 4).

Curiously, in testing LB and minimal medium cultures without a plasmid, we did not see a convincing difference in the levels of EIIA^Ntr^-P between *sixA^+^* and Δ*sixA* strains. However, we note that for these cultures, EIIA^Ntr^ was predominantly phosphorylated in the *sixA*^+^ strain, making it difficult to detect a further increase of EIIA^Ntr^ phosphorylation in the Δ*sixA* strain. At present, we do not understand why the cells with and without a plasmid showed different effects. Nevertheless, the results described above show that SixA affects PTS^Ntr^ phosphorylation. In particular, the absence of SixA can lead to hyperphosphorylation of EIIA^Ntr^.

### SixA modulates EIIA^Ntr^ phosphorylation through NPr.

In our working model for the Δ*sixA* growth defect, SixA-null strains have increased phosphorylation of one or more PTS^Ntr^ proteins. This in turn causes the putative potassium-proton antiporter YcgO to become more active, and the Δ*sixA* cells suffer from the consequences of YcgO overactivity (Fig. 2D) (13). Consistent with this model, we found that the Δ*sixA* and Δ*ptsN* strains have similar growth defects (Fig. 3B). The double deletion strain grew slightly more slowly than strains with either deletion alone, which accounts for the OD of the double deletion being slightly lower than the ODs of the single deletions.

According to a model proposed previously (13), unphosphorylated EIIA^Ntr^ inhibits YcgO, and slow growth results from loss of this inhibition. We reasoned that the absence of EI^Ntr^ or NPr, the first two components of the PTS^Ntr^ (Fig. 2D), would cause EIIA^Ntr^ to be fully unphosphorylated. In this case, eliminating the SixA phosphatase would have no effect on growth. Indeed, deletion of *sixA* had no effect on the growth of an NPr-null strain, as anticipated (Fig. 3C). However, we found that deletion of *sixA* still produces a growth defect in a strain lacking EI^Ntr^.

We interpret this unexpected result in the context of our model in which the growth defect arises from increased phosphorylation of the PTS^Ntr^. The fact that deleting *sixA* affected growth of an EI^Ntr^-null strain indicates that there is another source of phosphoryl groups for the PTS^Ntr^, which we hypothesize is the sugar phosphotransferase system (PTS^sugar^) (Fig. 3D). The PTS^sugar^ is a pathway homologous to the PTS^Ntr^ that facilitates carbohydrate uptake into the cell by phosphorylating transported carbohydrates (reviewed in references 16 and 17). Previous work has shown that EIIA^Ntr^ can be phosphorylated by PTS^sugar^ components (11, 18–21). This cross talk might be expected to occur during growth on non-PTS carbohydrates such as glycerol, which is characterized by high phosphorylation levels of PTS^sugar^ proteins, but not for growth on a PTS sugar such as glucose, for which PTS^sugar^ proteins are less phosphorylated (21, 22). We therefore hypothesized that growth on glucose would limit cross talk between the PTS^sugar^ and PTS^Ntr^. With this additional source of phosphorylation eliminated, we expected our original reasoning to apply, i.e., that deleting *sixA* should have no effect on growth in strains lacking either EI^Ntr^ or NPr. In agreement with this hypothesis, deleting *sixA* had no effect on the growth of either EI^Ntr^-null or NPr-null strains in glucose minimal medium (Fig. 3E). These observations support our model suggesting that the Δ*sixA* slow-growth phenotype requires phosphoryl group transfer through the PTS^Ntr^. Furthermore, the results presented in Fig. 3C and E imply that SixA modulates PTS^Ntr^ through NPr, since in the absence of NPr, the deletion of *sixA* has no effect.

To test whether SixA modulation of EIIA^Ntr^ phosphorylation depends on NPr, we overexpressed SixA in the presence or absence of NPr and monitored levels of EIIA^Ntr^ and EIIA^Ntr^-P. We observed significant EIIA^Ntr^ phosphorylation in the absence of NPr for cells growing on LB (Fig. 3F, lanes 1 and 3), which is consistent with cross talk to EIIA^Ntr^. Furthermore, we found that SixA expression had no observable effect on EIIA^Ntr^-P in an NPr-null strain (Fig. 3F, lanes 3 and 4), supporting a model in which SixA acts on NPr.

## DISCUSSION

### Targets of SixA phosphatase activity.

Only one target of the SixA phosphohistidine phosphatase has been reported to date: E. coli ArcB. The interaction between these proteins was discovered through a screen for inhibitors of OmpR phosphorylation by the ArcB histidine phosphotransfer (HPt) domain (6). Further support was provided by *in vitro* data showing that SixA dephosphorylates ArcB and by *in vivo* results indicating that deleting *sixA* affects expression of an ArcA-dependent *sdh* transcriptional reporter under conditions of anaerobic respiration (6, 7). However, our experiments with a different ArcA-dependent reporter growing anaerobically on nitrate did not show any evidence that SixA modulates ArcA phosphorylation (Fig. 1E), and another study failed to see an effect of SixA on ArcA-regulated transcription under conditions of microaerobic growth (23). Thus, while the *in vitro* results reported in references 6 and 7 indicate that SixA can function as a phosphohistidine phosphatase, the role of SixA in modulating ArcB/ArcA signaling *in vivo* is unclear and requires further investigation.

The genetic and biochemical evidence presented here reveals a different—ArcB-independent—function for SixA: modulation of PTS^Ntr^ phosphorylation. As with other phosphotransferase systems, the activity of the PTS^Ntr^ depends on the phosphorylation states of its protein components. For example, many pathways are reportedly mediated by the unphosphorylated form of EIIA^Ntr^ (12, 13, 24–29; reviewed in reference 17). The phosphorylation states of the PTS^Ntr^ proteins depend on a balance between the rate of phosphoryl transfer into the system from phosphoenolpyruvate and the rate of dephosphorylation. However, the mechanism by which PTS^Ntr^ proteins are dephosphorylated has not been established. Whereas the PTS^sugar^ transfers phosphoryl groups to incoming sugars, providing a phosphoryl group sink for this system (Fig. 3D), no analogous sink for the PTS^Ntr^ has been reported. A recently proposed model for a global stress response pathway in Acinetobacter baumannii, a bacterium whose PTS^Ntr^ lacks an EIIA^Ntr^ protein, suggests a dephosphorylation pathway for NPr via phosphotransfer to a serine on another protein, GigB, which is subsequently dephosphorylated by a phosphoserine phosphatase (30). However, E. coli and many other bacteria that have a PTS^Ntr^ do not have GigB.

One possible path for PTS^Ntr^ dephosphorylation is through cross talk with the PTS^sugar^ (11, 18–21) (Fig. 3C), although the extent of this cross talk in wild-type bacteria is unknown. It is also possible that the PTS^Ntr^ can be dephosphorylated by spontaneous hydrolysis, due to the intrinsic instability of phosphohistidines of the phosphorylated PTS^Ntr^ proteins (31). Our work reveals a different mechanism that is mediated by the SixA phosphatase. On the basis of our observation that NPr is required for SixA to decrease EIIA^Ntr^ phosphorylation (Fig. 3F), we propose that SixA removes phosphoryl groups from the PTS^Ntr^ via NPr. Furthermore, since phosphohistidine phosphatase activity has been demonstrated *in vitro* for SixA, we hypothesize that SixA directly dephosphorylates NPr-P.

We were led to the observation that SixA modulates the PTS^Ntr^ through our discovery of a growth defect for the Δ*sixA* mutant and through our suppressor analysis, which pointed to a connection with the growth defect associated with the Δ*ptsN* mutant. We note, however, that we do not yet understand the mechanism underlying this growth defect or its suppression by Δ*ycgO*.

### Modulators of PTS^Ntr^ activation.

PTS^Ntr^ activation is influenced by both nitrogen and carbon metabolism. In particular, EI^Ntr^ is inhibited by glutamine and may be stimulated by α-ketoglutarate (32–34). In addition, EIIA^Ntr^ phosphorylation is influenced by PTS sugars through cross talk from the PTS^sugar^ in at least some genetic backgrounds (19–21). Such cross talk could account for our observation that the severity of the Δ*sixA* growth defect depends on the carbon source. Non-PTS carbon substrates such as glycerol, maltose, and acetate produce pronounced Δ*sixA* growth defects that are easily observable as small colonies on solid media. Growth on the PTS sugar glucose, however, yields a subtle growth defect that we could detect only with optical density measurements. These phenotypes suggest a model in which, during growth of E. coli Δ*sixA* on non-PTS substrates, increased phosphorylation of the PTS^sugar^ increases phosphorylation of the PTS^Ntr^ through cross talk, thereby exacerbating the Δ*sixA* slow-growth phenotype. In the context of this model, SixA is important for limiting cross talk from the PTS^sugar^ in wild-type E. coli. However, it is also possible that the dependence of the Δ*sixA* slow-growth phenotype on carbon source reflects differences in phosphoenolpyruvate levels.

SixA may also provide an additional point of control for the PTS^Ntr^, since factors affecting SixA expression or activity would modulate PTS^Ntr^ activation. For example, SixA protein levels may increase in response to cell envelope stress since *sixA* is a member of the σ^E^ regulon (35–38). Several examples of interactions between the PTS^Ntr^ and the cell envelope have been identified (39–41). Thus, increased dephosphorylation of the PTS^Ntr^ by SixA could play an important role in the cell envelope stress response.

### Concluding remarks.

SixA is a well-conserved protein found in proteobacteria, actinobacteria, and cyanobacteria (5). Gene expression and proteome analyses have implicated SixA in biofilms of E. coli and Acidithiobacillus ferrooxidans (42–45), in nutrient limitation-induced phenotypes of Legionella and Salmonella (46–48), and macrophage infection with Salmonella (38). The roles of SixA in these contexts were interpreted to involve oxygen limitation, ArcB, or other two component systems but may instead involve SixA regulation of phosphotransferase systems. Does SixA function as a PTS^Ntr^ modulator in other bacterial species? Are there other targets of SixA? Our understanding of the roles of phosphatases in modulating histidine phosphorylation is in its early stages in bacterial and eukaryotic systems alike.

## MATERIALS AND METHODS

Strains, plasmids, and primers used in this study are listed in Tables 1, 2, and 3, respectively.

**TABLE 1:** Strains used in this study

Strain	Relevant genotype	Reference, source, or construction
MG1655	F^−^ λ^−^ *ilvG rfb-50 rph-1*	E. coli Genetic Stock Center (CGSC no. 7740)
JW2337	Δ*sixA*::(FRT-*kan*-FRT)	51
JES12	MG1655 Δ*sixA*::(FRT-*kan*-FRT)	P1_vir_(JW2337) × MG1655
JES13	MG1655 Δ*sixA*::(FRT)	JES12 treated with pCP20
JW4364	Δ*arcA*::(FRT-*kan*-FRT)	51
JW5536	Δ*arcB*::(FRT-*kan*-FRT)	51
JES47	MG1655 Δ*arcA*::(FRT-*kan*-FRT)	P1_vir_(JW4364) × MG1655
JES48	MG1655 Δ*arcA*::(FRT-*kan*-FRT) Δ*sixA*::(FRT)	P1_vir_(JW4364) × JES13
JES49	MG1655 Δ*arcB*::(FRT-*kan*-FRT)	P1_vir_(JW5536) × MG1655
JES50	MG1655 Δ*arcB*::(FRT-*kan*-FRT) Δ*sixA*::(FRT)	P1_vir_(JW5536) × JES13
JES53	MG1655 Δ*arcA*::(FRT)	JES47 treated with pCP20
PK9483	MG1655 *lacZ*::(*kan* P*_*icdA*_*(-58GGTGA-54)-*lacZ*)	9
JES252	MG1655 *lacZ*::(*kan* P*_*icdA*_*(-58GGTGA-54)-*lacZ*)	P1_vir_(PK9483) × MG1655
JES253	MG1655 *lacZ*::(*kan* P*_*icdA*_*(-58GGTGA-54)-*lacZ*) Δ*sixA*::(FRT)	P1_vir_(PK9483) × JES13
JES254	MG1655 *lacZ*::(*kan* P*_*icdA*_*(-58GGTGA-54)-*lacZ*) Δ*arcA*::(FRT)	P1_vir_(PK9483) × JES53
JW5184	Δ*ycgO*::(FRT-*kan*-FRT)	51
JES30	MG1655 Δ*ycgO*::(FRT-*kan*-FRT)	P1_vir_(JW5184) × MG1655
JES31	MG1655 Δ*ycgO*::(FRT-*kan*-FRT) Δ*sixA*::(FRT)	P1_vir_(JW5184) × JES13
JW3171	Δ*ptsN*::(FRT-*kan*-FRT)	51
JES208	MG1655 Δ*ptsN*::(FRT-*kan*-FRT)	P1_vir_(JW3171) × MG1655
Z501	*ptsN*::(*ptsN*-3×FLAG FRT-*kan*-FRT)	15
JES211	MG1655 *ptsN*::(*ptsN*-3×FLAG FRT-*kan*-FRT)	P1_vir_(Z501) × MG1655
JES212	MG1655 *ptsN*::(*ptsN*-3×FLAG FRT-*kan*-FRT) Δ*sixA*::(FRT)	P1_vir_(Z501) × JES13
JES264	MG1655 Δ*sixA*::(FRT) Δ*ptsN*::(FRT-*kan*-FRT)	P1_vir_(JW3171) × JES13
JW2408	Δ*ptsP*::(FRT-*kan*-FRT)	51
JW3173	Δ*ptsO*::(FRT-*kan*-FRT)	51
JES189	MG1655 Δ*ptsP*::(FRT-*kan*-FRT)	P1_vir_(JW2408) × MG1655
JES190	MG1655 Δ*ptsP*::(FRT-*kan*-FRT) Δ*sixA*::(FRT)	P1_vir_(JW2408) × JES13
JES185	MG1655 Δ*ptsO*::(FRT-*kan*-FRT)	P1_vir_(JW3173) × MG1655
JES186	MG1655 Δ*ptsO*::(FRT-*kan*-FRT) Δ*sixA*::(FRT)	P1_vir_(JW3173) × JES13
JES270	MG1655 Δ*ptsO*::(FRT-*cat*-FRT)	See Materials and Methods
JES271	MG1655 *ptsN*::(*ptsN*-3×FLAG FRT-*kan*-FRT) Δ*ptsO*::(FRT-*cat*-FRT)	P1_vir_(JES270) × JES211
E. cloni Replicator	F^−^ *mcrA* Δ(*mrr*-*hsdRMS*-*mcrBC*) *endA1 recA1* φ80d*lacZ*ΔM15 Δ*lacX74 araD139* Δ(*ara*, *leu*) *7697 galU galK rpsL nupG tonA* (*attL araC*-P*_*BAD*_*-*trfA250 bla attR*) λ^−^	Lucigen Corporation
PIR2	F^−^ Δ*lac169 rpoS*(Am) *robA1 creC510 hsdR514 endA recA1 uidA*(Δ*MluI*)::*pir*	Invitrogen
TOP10	F^−^ *mcrA* Δ(*mrr*-*hsdRMS*-*mcrBC*) *endA1 recA1* φ80*lacZ*ΔM15 Δ*lacX74 araD139* Δ(*ara-leu*) *7697 galU galK rpsL nupG*	Invitrogen

**TABLE 2:** Plasmids used in this study

Plasmid	Relevant genotype	Reference or construction
pTrc99a	*lacI*^q^ *P_*trc*_*, MCS from pUC18, *rrnB*(Ter) *bla ori* pMB1	59
pSixA (pJS17)	pTrc99a *sixA*	NcoI-sixA-F + HindIII-sixA-R
pSixA(H8A) (pJS21)	pTrc99a *sixA*(H8A)	sixA-dead-F + sixA-deadCRIM-R2
pMG91 (pSMART)	pSMART VC BamHI *oriV ori2 repE parABC cat*	Lucigen Corporation
pYcgO (pJS15)	pSMART *ycgO*	EcoRI-PcvrA-F + HindIII-cvrA-R
pBR322	*rop*, *tet*, *bla*, *ori* pMB1	60
pEB52	pTrc99a with NcoI restriction site removed	61
pEIIA^Ntr^ (pJS37)	pEB52 *ptsN*-3×FLAG	EcoRI-ptsN-F + HindIII-ptsN-3×FLAG-R2
pRL27	*ori*R6Kγ *aph oriT* P*_*tetA*_*-*tnp*	54
pCP20	λ*c*I857(ts) λ*p*_R_-FLP *repA101*(ts) *oriR101 bla cat*	50
pEL8	pCP20 Δ*cat*	62
pKD3	*ori*R6Kγ *bla* FRT*-cat-*FRT	63

**TABLE 3:** Primers used in this study

Primer	Sequence	Resulting construct or purpose
NcoI-sixA-F	GCATGCCATGGGCGGTGCAATATGCAAG	pSixA
HindIII-sixA-R	GCATCGAAGCTTGGAACTCATCAGATAG	pSixA
sixA-dead-F	GGCGACGCAGCCCTCGATG	pSixA(H8A)
sixA-deadCrim-R2	CGCACGCATGATAAAAACTTG	pSixA(H8A)
EcoRI-PcvrA-F	CGCTCGAATTCTGATCGGTACACCGTGGATG	pYcgO
HindIII-cvrA-R	CCAGTAAGCTTCAACCGGCGATAGATGCTTC	pYcgO
EcoRI-ptsN-F	GCATGGAATTCTGAGTGGGCAGGTTCTTAG	pEIIA^Ntr^
HindIII-ptsN-3×FLAG-R2	CCAGTAAGCTTCAGCTCCAGCCTACATTAC	pEIIA^Ntr^
psyn-u1	CCTGACGGATGGCCTTTTTG	PCR-amplify FRT-*cat*-FRT
oriR6Kseqprim1	GACACAGGAACACTTAACGGC	PCR-amplify FRT-*cat*-FRT
pBR322-seq-For	CCTGCTCGCTTCGCTACTTG	Sequence insertions from plasmid library screen
pBR322-seq-Rev	GCGATATAGGCGCCAGCAAC	Sequence insertions from plasmid library screen

### Growth conditions.

Rich medium consisted of LB Miller medium (49) (containing [per liter] 10 g tryptone, 5 g yeast extract, and 10 g NaCl). Defined medium consisted of minimal A medium (49) (containing [per liter] 10.5 g K_2_HPO_4_, 4.5 g KH_2_PO_4_, 1 g (NH_4_)_2_SO_4_, and 0.5 g Na citrate dihydrate) with 0.2% of the indicated carbon source and 1 mM MgSO_4_ added after autoclaving. Minimal medium plates contained 1.5% agar. Bacteria were grown at 37°C, except during some steps of strain construction, when cultures were grown at 30°C to maintain temperature-sensitive plasmids. The final concentrations of the antibiotics ampicillin, kanamycin, and chloramphenicol were 50 to 100, 25 to 50, and 12 to 25 µg/ml, respectively. Plasmids containing *sixA* or *ptsN* were used without IPTG (isopropyl-β-d-thiogalactopyranoside) induction and thus expressed the corresponding proteins from the basal activity of the *trc* promoter.

For anaerobic growth, minimal medium containing 0.2% glucose and 0.1% Casamino Acids was inoculated with a single colony and transferred to an anaerobic chamber, and the culture was grown overnight to stationary phase. The medium for the following day's cultures, which was the same as that used for the overnight cultures but also contained 40 mM KNO_3_, was also put in the anaerobic chamber at that time. The overnight cultures were used to inoculate the medium containing KNO_3_ (1:500 dilution of the inoculum) and were grown for 7 to 8 doublings anaerobically.

### Strain construction.

Phage transduction was performed with P1_vir_ as described in reference 49. FLP recombination target (FRT)-flanked kanamycin resistance cassettes were excised from the genome with FLP recombinase expressed from pCP20 (50). Gene deletions from the Keio collection (51) were confirmed by PCR.

To construct JES270, a chloramphenicol-resistant and kanamycin-sensitive Δ*ptsO* strain, a PCR fragment containing FRT-*cat*-FRT was amplified from pKD3 with primers psyn-u1 and oriR6kseqprim1. The resulting DNA segment was then electroporated into strain JES185/pEL8, and cells were selected on 12 μg/ml chloramphenicol to exchange FRT-*kan*-FRT with FRT-*cat*-FRT. The resulting strain was confirmed to be kanamycin and ampicillin sensitive, indicating loss of both the kanamycin resistance gene and plasmid pEL8.

### Plasmid construction.

The insertions in all engineered plasmids were verified to be correct by DNA sequencing.

To construct *sixA* expression plasmid pSixA, primers NcoI-sixA-F and HindIII-sixA-R were used to amplify the *sixA* gene from MG1655 genomic DNA. The resulting DNA segment was digested with NcoI and HindIII and cloned into pTrc99a, which was digested with the same enzymes. A plasmid expressing the H8A *sixA* mutant was constructed by site-directed mutagenesis as follows: plasmid pSixA was amplified with primers sixA-dead-F and sixA-deadCRIM-R2, the ends were phosphorylated with T4 polynucleotide kinase, and the resulting DNA was circularized by blunt-end ligation with T4 DNA ligase, generating pSixA(H8A).

To construct the *ycgO* expression plasmid pYcgO, the *ycgO* gene and roughly 500 bp of upstream DNA were amplified from MG1655 genomic DNA with primers EcoRI-PcvrA-F and HindIII-cvrA-R. The resulting PCR product was digested with EcoRI and HindIII and ligated into EcoRI- and HindIII-digested pMG91, a derivative of the single-copy plasmid pSMART.

The plasmid for FLAG-tagged *ptsN* expression was constructed as follows. The *ptsN*-3×FLAG sequence from Z501 genomic DNA was amplified with primers EcoRI-ptsN-F and HindIII-ptsN-3×FLAG-R2. The resulting PCR product was cloned into EcoRI- and HindIII-digested pEB52, a derivative of pTrc99a, generating pEIIA^Ntr^.

### Growth curves.

LB Miller medium was inoculated with a single colony and grown to stationary phase with aeration in a roller drum. The overnight culture was then used to inoculate 50 ml of medium with a 1:1,000 dilution, and the flasks were aerated on a shaker. For each time point, three aliquots were removed and the optical density at 600 nm (OD_600_) of each was measured. For determination of the wild-type strain’s growth curve in glycerol minimal medium (Fig. 1A), the exponential-phase and stationary-phase measurements were obtained from two separate experiments. The data for the growth curves presented in Fig. 2A and Fig. 3B were collected in the same experiment, so the curves for MG1655 and JES13 are identical.

### Optical density endpoint assay.

Our initial experiments with endpoint assays revealed that cells in some cultures were aggregating, which was manifested by the flocculation and sedimentation of standing cultures and was also visible by microscopy. We suspect that spontaneous activation of antigen 43, which is subject to random phase variation and causes autoaggregation when expressed (52, 53), was responsible, since deletion of *flu* eliminated this flocculation phenotype. The flocculating cultures were not used for endpoint assays because they produced lower optical densities than nonflocculating cultures.

LB Miller cultures were inoculated with single colonies and grown overnight to stationary phase with aeration in a roller drum. These cultures were then left standing on the benchtop at room temperature for at least 1 h to monitor for flocculation of the culture. Those tubes that did not show flocculation were then used to inoculate 2-ml cultures of minimal medium (1:1,000 dilution), which were grown with aeration in a roller drum. Incubation times were chosen to catch wild-type cultures in exponential phase.

### β-Galactosidase assays.

β-Galactosidase assays were performed essentially as described previously (49). To ensure that protein synthesis was stopped at the appropriate times, 100 µg/ml streptomycin was added to cultures after they were removed from the anaerobic chamber and placed on ice.

### Genetic screens.

For transposon mutagenesis, electrocompetent Δ*sixA* JES13 was transformed with pRL27, which contains a hyperactive Tn*5* transposase (54). After electroporation and recovery in SOC medium (containing [per liter] 20 g tryptone, 5 g yeast extract, 0.5 g NaCl, with 20 mM MgSO_4_ and 0.4% glucose added after autoclaving) for over 1 h, the cells were incubated in SOC medium with kanamycin for 6 h to select for mutants. Aliquots were then plated on maltose minimal medium agar, and >16,000 colonies were screened to identify suppressor mutants. Genomic DNA sequences flanking the sites of transposon insertions were identified as described previously (55, 56).

For the multicopy suppressor screen, JES13 was transformed with a plasmid library prepared from MG1655 genomic DNA fragments cloned into pBR322 (57) and was screened on minimal agar medium containing several different supplements: (i) glucose with 0.1% Casamino Acids and 15 to 30 mM procaine HCl, (ii) maltose, (iii) glycerol, and (iv) acetate. Over 11,000 colonies were screened to identify plasmids that suppress slow growth. Plasmids were isolated, retransformed into JES13, and tested on maltose minimal medium agar plates to confirm that the plasmid suppressed the slow-growth phenotype. Insert sequences were determined by sequencing with primers pBR322-seq-For/pBR322-seq-Rev.

### Potassium measurements.

LB Miller overnight cultures inoculated from single colonies were used to inoculate 2-ml cultures of glycerol minimal medium, which were grown overnight in a roller drum to optical densities of approximately 0.2. Cultures were harvested by centrifugation through a layer of a 2:1 (vol/vol) mixture of dibutyl phthalate and dioctyl phthalate following the procedure described in reference 13. The cell pellet was suspended in 0.1 ml 1 N nitric acid and digested for 1 to 3 days. The samples were then diluted with 0.95 ml diluent (2% nitric acid, 0.5% HCl) and centrifuged at 13,000 × *g* for 30 min. One milliliter of supernatant was removed and mixed with 2 ml more diluent. ^39^K was measured in the samples and in standard reference controls using an Agilent 7900 inductively coupled plasma mass spectrometer (ICP-MS). The limit of quantification was determined to be 0.1 mg/liter. Potassium content was determined as nanomoles of potassium per OD_600_ of the culture prior to harvesting.

### EIIA^Ntr^ phosphorylation assay.

EIIA^Ntr^ phosphorylation assays were performed similarly to those described previously (14, 15, 58). Native gels were prepared with 10% 37.5:1 acrylamide and 375 mM Tris HCl (pH 8.8) and cast using a Mini-Protean II system (Bio-Rad). Cultures were grown overnight in LB Miller medium containing ampicillin to stationary phase. Cell pellets from 0.5 ml or 1 ml of culture were suspended in 0.4 ml or 0.8 ml of loading dye (10% glycerol, 40 mM glycine, 5 mM Tris HCl, 0.005% bromophenol blue, pH 8.8) for the experiments whose results are presented in Fig. 3A or Fig. 3F, respectively. The cells were then sonicated on ice and centrifuged to pellet cell debris. Samples were run at 4°C with a running buffer consisting of 25 mM Tris, 192 mM glycine (pH 8.3) until the dye reached the end of the gel.

After electrophoresis, proteins were transferred to a 0.45-μm-pore-size Immobilon-P polyvinylidene difluoride (PVDF) membrane (Millipore) using a semidry transfer apparatus and transfer buffer consisting of 20% methanol, 25 mM Tris, and 192 mM glycine (pH 8.3). The membrane was blocked with 5% milk–Tris-buffered saline with Tween 20 (TBST) (containing [per liter] 8 g NaCl, 0.38 g KCl, 3 g Tris base, 500 μl Tween 20, pH 7.4). Anti-FLAG mouse antibody (GeneTex; catalog no. GTX82562), digital anti-mouse horseradish peroxidase (HRP) antibody (Kindle Biosciences), and a KwikQuant Western blot detection kit and imager (Kindle Biosciences) were used to probe and image membranes.
